# Bioconversion of bread waste into high-quality proteins and biopolymers by fermentation of archaea *Haloferax mediterranei*

**DOI:** 10.3389/fmicb.2024.1491333

**Published:** 2024-12-24

**Authors:** Razan Unis, Rima Gnaim, Mrinal Kashyap, Olga Shamis, Nabeel Gnayem, Michael Gozin, Alexander Liberzon, Jallal Gnaim, Alexander Golberg

**Affiliations:** ^1^Department of Environmental Studies, Porter School of Environment and Earth Sciences, Tel Aviv University, Tel Aviv, Israel; ^2^The Triangle Regional R&D Center (TRDC), Kfar Qari, Israel; ^3^Faculty of Exact Sciences, School of Chemistry, Tel Aviv University, Tel Aviv, Israel; ^4^Center for Advanced Combustion Science, Tel Aviv University, Tel Aviv, Israel; ^5^Center for Nanoscience and Nanotechnology, Tel Aviv University, Tel Aviv, Israel; ^6^School of Mechanical Engineering, Tel Aviv University, Tel Aviv, Israel

**Keywords:** microbial fermentation, *Haloferax mediterranei*, bread waste, biopolymer, poly(3-hydroxybutyrate-co-3-hydroxyvalerate)

## Abstract

The valorization of bread waste into high-quality protein and biopolymers using the halophilic microorganism *Haloferax mediterranei* presents a sustainable approach to food waste management and resource optimization. This study successfully coproduced protein and poly(3-hydroxybutyrate-co-3-hydroxyvalerate) (PHBV) biopolymer with a biomass content of 8.0 ± 0.1 g L^−1^ and a productivity of 11.1 mg L^−1^ h^−1^. The fermentation process employed 3.0% w/v of enzymatically hydrolyzed bread waste. The amino acid profile of the cell biomass revealed a total content of 358 g kg^−1^ of biomass dry weight (DW), including 147 g kg^−1^ DW of essential amino acids. The protein quality, assessed through *in-vitro* enzyme digestion, indicated a high-quality protein with a digestibility value of 0.91 and a protein digestibility-corrected amino acid score (PDCAAS) of 0.78. The PHBV biopolymer component (36.0 ± 6.3% w/w) consisted of a copolymer of 3-hydroxybutyrate and 3-hydroxyvalerate in a 91:9 mol% ratio. This bioconversion process not only mitigates food waste but also generates valuable biomaterials.

## Introduction

The global population’s rapid growth and the consequential rise in starvation, malnutrition, and related diseases worldwide have intensified the need for adequate and continuous sources of nutrients (Bratosin et al., 2021). Meeting the growing demand for protein-rich foods through agriculture alone is challenging and complex (Fasolin et al., 2019); therefore, the search for alternative protein sources, such as microbial biomass, has been increasing (Al-Mudhafr, 2019).

Microorganisms have been employed for an extended period in producing food items with elevated protein content, including cheese and fermented soybean products (Upadhyaya et al., 2016). The selection of microorganisms for this purpose depends on multiple criteria, such as rapid growth on a wide range of appropriate substrates (Sharif et al., 2021). Additional measures include nutritional (e.g., energy value, protein content and yield, amino acid, and essential amino acid balance) and procedural aspects (e.g., the sort of culture, sterilization method, aeration, agitation, fermentation time, product isolation method, purification method, and the efficiency of the fermentation process) (Ravindra, 2000). The main strategies regarding a substrate needed to produce microbial biomass containing high quality protein involve utilizing low-grade waste materials or a simple carbohydrate source (Dunuweera et al., 2021).

The archaea *Haloferax mediterranei* belongs to the extremely halophilic class of halobacteria and has numerous advantages for protein production, such as the ability to survive and grow in high salinities, thus reducing microbial contamination risks (Pacholak et al., 2021). *H. mediterranei* has rapid growth compared with related organisms and a broader substrate spectrum (Mironescu et al., 2004). It allows for adaptability to varying environmental conditions encountered during the fermentation process, encompassing changes in oxygen levels, temperature fluctuations, nutrient concentrations, and pH (Matarredona et al., 2020; Matarredona et al., 2021; García-Chumillas et al., 2024). In addition, *H. mediterranei* is sensitive to hypotonic media and can be efficiently lyzed in distilled water; therefore, using large quantities of organic solvents in the extraction process can be avoided (Giani and Martínez-Espinosa, 2020; Simó-Cabrera et al., 2021).

Halophiles have been investigated for functional protein production, specifically for enzymes such as pullulanases, proteases, lipases, hydrolases, amylases, and DNases (De Lourdes Moreno et al., 2013), PHA-associated regulatory protein PhaR, granule-associated protein, PhaP (Zhang et al., 2018), and extracellular polymeric substances (EPS) (Cui et al., 2017). In addition, various studies have confirmed the suitability of halophiles as efficient protein bioreactors (Lillo and Rodriguez-Valera, 1990; Simó-Cabrera et al., 2021; Giani et al., 2021; Rodrigo-Baños et al., 2021; Martínez-Espinosa, 2024).

In addition, *H. mediterranei* is a versatile intracellular polyhydroxyalkanoate (PHA) producer that has gained attention due to its potential to synthesize PHAs from simple and inexpensive carbon sources (Koller, 2015). PHAs are biopolymers that exhibit mechanical and thermal characteristics analogous to traditional plastics such as polyethylene and polypropylene. Among the interesting products produced by extremely halophilic archaea are poly(3-hydroxybutyrate) (PHB) and poly(3-hydroxybutyrate-co-3-hydroxyvalerate) (PHBV) (Gonzalez and Winterburn, 2022). PHB is characterized by its hardness and brittleness; it has a melting point closely approaching its degradation temperature, thereby limiting its utility due to a narrow temperature processing range (Ferre-Guell and Winterburn, 2018). However, PHBV is less crystalline, more flexible, and highly processable (Sato et al., 2021). Thus, it is gaining increasing importance in food packaging, agriculture, and biomedical applications such as tissue engineering scaffold fabrication, wound healing, and medical implant development (Cai et al., 2021). Interestingly, *H. mediterranei* is among the few microorganisms that synthesize PHBV from a simple and cheap carbon source without supplementing the 3-hydroxy valeric acid precursor (Zhao et al., 2013).

Numerous investigations on the production of PHA using *H. mediterranei* have focused on harnessing industrial and agricultural byproducts, such as extruded rice bran (Huang et al., 2006), vinasse (Bhattacharyya et al., 2012), rice-based ethanol stillage (Bhattacharyya et al., 2015), cheese whey (Pais et al., 2016), olive mill wastewater (Alsafadi and Al-Mashaqbeh, 2017), molasses wastewater (Cui et al., 2017), macroalgal biomass (Ghosh et al., 2019), ricotta cheese exhausted whey (Raho et al., 2020), date palm fruit waste (Alsafadi et al., 2020), candy industry waste (Simó Cabrera et al., 2024), and bread waste (Montemurro et al., 2022). However, the industrial production of PHA is still hindered by the costly feed materials (Koller et al., 2005).

The coproduction of PHA using various microorganisms (Li et al., 2017) with other valuable chemicals has been demonstrated; these include amino acids (Gu et al., 2013), enzymes (Shamala et al., 2012), alcohols (Xin et al., 2007), molecular hydrogen (Singh et al., 2013), biosurfactants (Rashid et al., 2015), exopolysaccharides (Cui et al., 2017), and carotenoids (Kumar et al., 2018). Umesh et al. investigated the production of PHA by *Bacillus subtilis* and proteins by *Saccharomyces cerevisiae* utilizing *Carica papaya* waste (Umesh et al., 2017). *Cupriavidus necator* cells were evaluated as a source of protein and used to recover PHA granules simultaneously (Chee et al., 2019). However, the coproduction of PHA and protein has not been investigated.

The main strategy regarding a substrate used to produce microbial biomass is to consider low-grade waste material (Carranza-Méndez et al., 2022). Bread waste (BW) is regarded as a potential carbon/nitrogen source. The global annual bread production is >100 million tonnes (Narisetty et al., 2021). Owing to a short shelf-life and the overproduction of bread, approximately 10% (~ 10 million tonnes) of the bread produced globally is discarded; it amounts to around 24 million slices of bread every day, representing 27–31% of the total food waste mass (Montemurro et al., 2022). In the UK, the largest bread consumer in Europe, approximately 0.3 million tonnes of bread is wasted annually (Jung et al., 2022). In Israel and the United States, within the grain and legume category, the waste rate stands at approximately 14% (0.17 million tonnes) and 25% (0.31 million tonnes), respectively (Philip et al., 2017). Disposal of BW without its valorization could result in the loss of resources (Jung et al., 2022). Hydrogen, ethanol, lactic acid, succinic acid, lipids, and PHA are examples of high-value products generated by the microbial fermentation of BW (Montemurro et al., 2022). Therefore, BW can potentially be utilized as a valuable and sustainable carbon/nitrogen source that would contribute to a sustainable coproduction of PHBV and proteins while simultaneously addressing waste reduction and resource optimization challenges.

The current research focused on assessing the potential of *H. mediterranei* as a versatile microorganism capable of simultaneously producing PHBV and proteins. This was achieved by utilizing the enzymatic hydrolysate of BW as a nutrient-rich carbon/nitrogen source, along with red sea salt as a comprehensive growth medium. Specifically, the research aimed to (i) investigate the key factors affecting the growth of *H. mediterranei* under various conditions, (ii) determine the chemical composition of the resulting archaea, including protein, PHBV polymer, ash content, macroelements, trace elements, lipids, and carbohydrates, and (iii) determine the digestibility characteristics of the generated proteins.

## Materials and methods

### Materials

Yeast extract was obtained from Thermo Fisher Scientific (Difco^™^, Israel); it consisted (w/w) of 34.76% C, 9.30% N, 6.31% H, and 0.54% S with a C:N ratio of 3.74. Red sea salt was obtained from Aquazone Ltd. (Israel) and contained (g kg^−1^): Na 358.9, Cl 553.9, Mg 37.4, S 25.7, Ca 12.3, K 11.4, Sr. 0.234, B 0.126, F 0.037, I 0.002, and other minor trace elements. The trace elements solution was prepared as follows: (mg L^−1^) ZnSO_4_·7H_2_O, 100; H_3_BO_3_, 300; CoCl_2_·6H_2_O, 200; CuSO_4_, 6; NiCl_2_·6H_2_O, 20; Na_2_MoO_4_·2H_2_O, 30; MnCl_2_·2H_2_O, 25 (Koller et al., 2008). The *α*-amylase enzyme from *Bacillus amyloliquefaciens* (≥250 U g^−1^), amyloglucosidase enzyme from *Aspergillus niger* (≥260 U mL^−1^), and alcalase® protease enzyme from *Bacillus licheniformis Subtilisin A* (≥2.972 U mL^−1^) were purchased from Sigma-Aldrich (Israel). Thirteen different BW samples and their mixtures were collected from local restaurants and bakeries (Kfar Qara’, Israel).

### Dry weight, ash content, and elemental analysis of BW

#### Dry weight of BW

Ten grams of fresh BW samples were cut into 1–2 cm pieces and were dried in an air oven (Carbolite, Israel) at 105°C for 3 days. The dried samples were ground to a fine powder using a blender (Gold line, Israel), then weighed and stored in closed containers at −20°C until use.

#### Ash content of BW

One gram of dry powder of BW was put in a pre-weighed crucible. The crucible containing the BW sample was subjected to heating at 550°C for 5 h. Next, the crucible with the remaining ash was cooled at 25°C and weighed, and the ash content was calculated (Gnaim et al., 2023).

#### Elemental analysis of BW

CHNS elemental analysis of the dry BW samples was determined utilizing a Thermo Scientific^™^ FLASH 2000 CHNS/O Analyzer (Technion, Israel). The determination of both the macroelements and trace elements involved the utilization of a PerkinElmer NexION 2000 inductively coupled plasma mass spectrometer (ICP-MS). This analysis was conducted at the Field Service Lab Central District (Hadera, Israel).

### Enzymatic hydrolysis of BW

Enzymatic hydrolysis of dry BW samples was carried out in three steps (Hudečková et al., 2017). First, 10 g of BW samples were homogenized with 100 mL of distilled water. The pH of the slurry was adjusted to 6.0; then, 4 mL of thermostable *α*-amylase (250 U g^−1^) was added. The mixture was kept at 80°C for 3 h with magnetic stirring at 150 rpm. The liquefaction was curtailed by freezing the mixture at −20°C for 12 h. In the second hydrolysis step, saccharification, the pH of the liquefied suspension was adjusted to 4.2, and the saccharification was performed in liquefied suspension by adding 4 mL amyloglucoamylase (260 U mL^−1^) at 60°C for 90 min. Heating the enzyme at 80°C for 5 min resulted in its inactivation, followed by subsequent cooling of the mixture to room temperature. Next, 1 mL of endopeptidase alcalase (2.972 U mL^−1^) was used in the third step after pH adjustment to 8.0, followed by heating at 80°C for 24 h. The enzyme’s activity was nullified by subjecting the suspension to heating at 100°C for 5 min. Finally, the mixture was filtered and stored at 4°C until use.

### Glucose determination

The concentration of glucose in the hydrolysate was determined using the D-glucose Assay Kit (GOPOD, Megazyme, Ireland), following the manufacturer’s protocol. Briefly, 0.1 mL of the hydrolysate sample was mixed with 3.0 mL of the GOPOD reagent, which contains glucose oxidase and peroxidase. The reaction between D-glucose and the reagents forms a colored compound, which was incubated at 40–50°C for 20 min. The absorbance of the resulting solution was measured at 510 nm using a spectrophotometer (Infinite M Plex Elisa, Tecan, Austria). A reagent blank was used as a reference, and a glucose standard solution was prepared to create a calibration curve for accurate quantification. The glucose concentration in the samples was calculated based on the absorbance relative to the glucose standard.

### Archaea strain and red sea salt medium preparation

*H. mediterranei* (ATCC 33500, CCM 3361) from the DSMZ (DSM 1411) culture collection was used for strain activation and culture. The following medium was employed for all *H. mediterranei* cultivation experiments under different conditions: 9.0–20.0% w/v of red sea salt powder, 0–24 mL L^−1^ of trace element solution, 0–0.07% w/v of NH_4_Cl, 0–0.06% w/v of KH_2_PO_4_, 0–5.5% w/v of glucose, 0–0.5% w/v of yeast extract, and 0–5.5% w/v of BW hydrolysate were added to 800 mL deionized water with a stirring rate of 300 rpm at 42°C for 3 h. Next, the solution was microfiltered under vacuum (Corning^®^ 500 mL, United States), and deionized water was added to complete the volume up to 1,000 mL. In the preparation of large volumes of medium (liters scale), the solution was autoclaved at 121°C for 30 min (Chauhan et al., 2020). Finally, the pH of the solution was adjusted (using 1 M NaOH or 1 M HCL solution) in the range of 2 to 13 and kept at 4°C until use.

### Cultivation of *Haloferax mediterranei* in 96-well plates

Cell pellets obtained from 2 to 24 μL of inoculum solution were resuspended in 176–198 μL of red sea salt medium in a 96-well plate, sealed with an adhesive plate sealer, and cultivated at 42°C for 120 h with shaking at 150 rpm. The culture microplate was shaken for 30 s and then placed into the multi-reader (Infinite M Plex Elisa, Tecan, Austria). The optical density was measured at 600 nm at 25°C at specific times of 0, 24, 48, 72, 96, and 120 h (5 replicates) with a cell-free supernatant serving as a blank. After 120 h, the cultivation solutions were transferred to 2 mL microcentrifuge tubes (Tarasons, India), and the cell biomass was collected by centrifugation (Neofuge 13R high-speed refrigerated benchtop centrifuge, China), operating at 13,000 rpm for 10 min. Next, the biomass was washed with 200 μL deionized water and dried at 60°C for 24 h. The resulting biomass was weighed and analyzed for PHBV and protein content using Fourier transform infrared (FTIR).

### Batch cultivation of *Haloferax mediterranei* in BW and red sea salt medium

Batch cultivation was conducted in culture flasks using a 100 mL solution containing 20 mL of *H. mediterranei* inoculum, 3 g of BW hydrolysate, and 20 g of red sea salt. The pH of the mixture was adjusted to 7.3, and then the mixture was cultured at 42°C for 72 h with constant shaking at 150 rpm. The culture flasks were sealed with aerated covers that allow oxygen to enter while keeping the environment controlled and preventing contamination. Following the cultivation, the cultures were subjected to centrifugation, washing, and drying at 60°C for 24 h. Finally, the dried biomass was weighed, and the components, including protein and PHBV, were examined using FTIR.

### Determination of ash content of *Haloferax mediterranei* biomass

Following the cultivation, the cultures were subjected to centrifugation, washing, and drying at 60°C for 24 h. Finally, the dried biomass was weighed. A dry, fine powder of cell biomass (200 mg) was put in a preweighted crucible and weighed again. The crucible containing the biomass sample was placed in a muffle furnace preheated at 550°C. The sample was kept in the furnace for 5 h. The crucible was carefully removed from the furnace, cooled to 100°C, and then placed in a desiccator to cool further. The crucible with the remaining ash was weighed, and the ash content was calculated.

### PHBV isolation from the cell biomass

A 200 mg of *H. mediterranei* biomass and a 10 mL chloroform were subjected to reflux at 62°C for 12 h. Next, the cooled mixture was filtered using a Whatman filter (no. 4, Macherey-Nagel, Germany). The off-white solid (non-PHBV cell mass) was collected, dried at 60°C for 12 h and weighed. In parallel, the supernatant, i.e., a PHBV/chloroform solution, was gradually poured into 20 mL methanol. Finally, the PHBV precipitate was isolated by centrifugation at 4,000 × g for 30 min, dried at 60°C for 24 h, weighed, and stored at −20°C. Analysis of amino acids in *H. mediterranei* biomass.

First, 100 mg of *H. mediterranei* biomass was placed in a glass tube and hydrolyzed with 5 mL of 6 N HCl solution and phenol at 110°C for 22 h. Another 100 mg aliquot of this sample was first oxidized with formic acid and hydrogen peroxide at 2–8°C for 16 h. Next, the oxidized samples were dried under vacuum and then hydrolyzed with 5 mL of 6 N HCl and phenol at 110°C for 22 h. Aliquots of the two hydrolysates were dried by a vacuum centrifuge and dissolved in an amino acid sample buffer. The hydrolysate solutions were sonicated, vortexed, and filtered using a 0.45 μm nylon filter. Next, 20 μL of the hydrolysate solutions were injected into the Biochrom 30+ Amino-Acid-Analyzer (AminoLab, Analytical Laboratory Services, Israel). The amino acids were separated on an ion exchange column (Biochrom H-1552), derivatized with ninhydrin after eluting from the column, detected at 570 and 440 nm, and quantified against a standard.

### Determination of the animal-safe accurate protein quality score

*H. mediterranei*’s total protein quality was assessed by evaluating their amino acid composition and *in-vitro* protein digestibility-corrected amino acid score (PDCAAS) using the Megazyme assay kit (Wicklow, Ireland, https://www.megazyme.com/). To 500 mg of milled biomass sample, 19 mL of 0.06 N HCl was added, and the mixture was incubated at 37°C for 30 min with shaking at 120 rpm. Next, 1 mL of pepsin solution was added, and the sample was incubated at 37°C for 1 h. The pH was brought to 7.4 with 2 mL of 1 M Tris–HCL buffer, followed by the addition of 200 μL of a trypsin-chymotrypsin mixture. The sample was vortexed and incubated at 37°C for 4 h with shaking at 120 rpm, then placed in a boiling water bath for 10 min. Next, the sample was vortexed, cooled to 25°C for 20 min, mixed with 1 mL of 40% trichloroacetic acid solution, incubated at 4°C overnight, and then centrifuged at 25°C for 10 min at 13,800 rpm. A 10-fold dilution in acetate buffer (50 mM, pH 5.5) was performed before the colorimetric assay. PDCAAS values were computed by utilizing the Megazyme Mega-CalcTM program (K-PDCAAS Mega-Calc). A standard curve derived from L-glycine used to plot the absorbance values recorded at 570 nm against L-glycine concentrations spanning from 0 to 1 mM was used to assess the primary amine concentration (*CI*) in unidentified samples. The concentration of primary amines (in mM) in the unknown samples was determined using Equation 1, in which *CI* represents the unknown primary amine concentration, *Y* corresponds to the absorbance, *B* denotes the *y*-intercept, and *A* represents the slope of the line.


(1) Y=A×CI+B

Equation 2 was used to calculate the primary amine concentration (*C_2_*) in the initial sample solution. In this equation, *CI* represents the concentration of primary amines in the samples after dilution, *D* stands for the dilution factor applied to the samples before amine measurement, 1.25 denotes the dilution factor associated with trichloroacetic acid, *W* represents the weight of the sample (g), and 0.5 signifies the nominal quantity (g).


(2) $$C_{2}=CI\times D\times 1.25\times \frac{0.5}{W}$$

Amino acid constants were employed to compute the adjusted primary amine concentration (*CN*) for the individual amino acids, as indicated in Equation 3. In this equation, *C2* represents the adjusted primary amine concentration in the initial sample solution (measured in mM), whereas proline, lysine, histidine, and arginine denote the concentrations of these respective amino acids in the original sample. The constants 2, 0.5, 0.2, and 0.2 are specific values associated with the corresponding amino acids.


(3) $$CN=C_{2}+\left(\frac{\mathrm{Pro}\times 2\times 10}{Lys\times 0.5\times 10}\right)+\left(\mathrm{His}\times 0.2\times 10\right)+\left(\arg\times 0.2\times 10\right)$$

*In-vitro* digestibility was determined utilizing Equation 4, which relies on established literature values for the rat model. To assess the corrected primary amine concentration (*CN*), the data fits were compared using a linear regression equation. In this equation, *X* denotes the corrected primary amine concentration for individual samples, *M* represents the slope of the regression line, *B* signifies the *y*-intercept, and 100 is the conversion factor used to convert percentages to grams.


(4) $$\mathrm{In}\;\mathrm{vitro}\;\mathrm{digestibility}=\frac{\left(M\times X+B\right)}{100}$$

The amino acid ratio and the identification of the limiting amino acids, expressed in grams per 100 grams of protein, were computed using the total crude protein values in the dry cell biomass, as demonstrated in Equation 5. The amino acid ratio within the sample was determined in accordance with the recommended values, as outlined in Equation 6. The *in-vitro* PDCAAS score was derived by multiplying the *in-vitro* digestibility obtained from Equation 4 by the limiting amino acid ratio (the smallest value) obtained from Equation 6.


(5) $$\mathrm{Amino Acid Ratio}\;\left[\frac{g}{100\;g\;\mathrm{Protein}}\right]=\frac{\mathrm{Amino Acid}\;\left[\frac{g}{100\;g\;\mathrm{protein}}\right]}{\mathrm{Crude Protein}\;\left[\%\right]}$$

(6) $$\mathrm{Amino Acid Ratio}=\frac{\mathrm{Sample}\;\left[\frac{\mathrm{mg}}{g\;\mathrm{Protein}}\;\right]\;}{\mathrm{Reference Sample}\;\left[\frac{\mathrm{mg}}{g\;\mathrm{Protein}}\;\right]\;}$$

### Analysis methods

#### Thermogravimetric and differential scanning calorimetry analysis

Thermogravimetric analysis (TGA) was employed to determine cell biomass degradation temperature (*T_d_*), along with the extracted PHBV and protein constituents. This involved subjecting the samples to a controlled heating regimen spanning the temperature range from 30 to 600°C, with a constant heating rate of 10°C min^−1^. Furthermore, the melting temperature (*T_m_*) was ascertained utilizing differential scanning calorimetry (DSC) over a temperature range of 30–600°C by employing a heating rate of 10°C min^−1^ (NETZSCH STA 449F5 STA449F5A-0214-M).

#### Fourier transform infrared analysis

FTIR spectra of the cell biomass, isolated PHBV, and isolated protein were recorded using a Thermo Scientific^™^ Nicolet^™^ iS50 FTIR Spectrometer, covering a spectral range from 400 to 4,000 cm^−1^ through 16 scan repetitions.

#### Nuclear magnetic resonance analysis of PHBV

The nuclear magnetic resonance (^1^H- and ^13^C-NMR) spectra were acquired on a Bruker 500 MHz NMR spectrometer while dissolving 15 mg of isolated PHBV in 0.5 mL of CDCl_3_ while heating at 50°C for 10 min.

#### Gel permeation chromatography analysis of PHBV

The gel permeation chromatography (GPC) analysis was carried out using an Agilent 1,260 Infinity II GPC system equipped with security Guard Cartridges GPC 4 × 3.0 mm ID 3/Pk XAJ0-9292, two GPC LF-804 columns, and one KF-803 column arranged in a sequence. The analysis was conducted in tetrahydrofuran at 35°C, following the methodology recently reported (Gnaim et al., 2023).

#### Gas chromatography–mass spectrometry

The characterization and quantification of the isolated PHBV were performed via gas chromatography–mass spectrometry (GC–MS) after the direct acid-catalyzed methanolysis of cell biomass, as described recently (Gnaim et al., 2021).

### Statistical analysis

Results were evaluated using a one-way ANOVA for the following variables: dry weight, ash content, and the C:N ratio of BW. Additionally, a two-way ANOVA with repeated measures was performed to investigate several abiotic factors (the pH, BW type, BW concentration, and red sea salt concentration) that affected the growth of *H. mediterranei*. Statistically significant differences were identified using Tukey multiple comparison tests using GraphPad Prism version 10, with significance indicated when *p <* 0.05.

## Results and discussion

### Collection and analysis of BW samples

Various BW samples (BW-1 to BW-13, Supplementary Figure S1) were collected from local restaurants and bakeries. Their commercial nutritional values, including carbohydrates, lipids, and proteins, are listed in Table 1. The carbohydrate contents in the BW samples ranged from 4.0 to 56.5% (w/w), lipids from 0 to 25.4% (w/w), and proteins from 9.1 to 27.5% (w/w). In addition, all BW samples were analyzed for their dry weight, ash content, C:N ratio, and the N-to-protein conversion factor. The corresponding values for dry weight ranged from 53.8 to 65.9% (w/w), ash content from 1.6 to 7.1% (w/w), C:N ratio from 7.1 to 23.4, and N-to-protein conversion factor from 4.9 to 7.9. The average N-to-protein conversion factor obtained for all BW samples in this study was 6.1, similar to that reported for wheat (5.8) (Fujihara et al., 2008). The protein concentration of BW was estimated by multiplying its nitrogen content, which was determined by elemental analysis, by the average N-to-protein conversion factor (6.1). Among the BW samples analyzed by one-way ANOVA and Tukey’s multiple comparisons test, BW-9 displayed the highest dry weight (65.6 ± 0.6% w/w) (significance, *p* < 0.0001). BW-2 exhibited the highest ash content (7.1 ± 0.4% w/w) (significance, *p* < 0.0001). BW-9 exhibited the highest C:N ratio (23.4 ± 0.1 w/w) (significance, *p* < 0.0001). BW-4 presented the highest N-to-protein conversion factor (7.9 ± 0.1). The large variability between different BW samples regarding the values of carbohydrates, lipids, proteins, dry weight, ash content, and the C:N ratio could result from differences in moisture content, mineral composition, protein composition, and nitrogen content in the BW samples. However, all BW samples were found to be rich in nitrogen, i.e., having a C:N ratio from 7.1 to 23.4, which is an essential factor for the growth of *H. mediterranei* and is suitable for microbial protein production.

**Table 1:** The carbohydrates (% w/w), lipids (% w/w), proteins (% w/w), dry weight (DW, % w/w of fresh weight), ash content (AC, % w/w of DW), CNS elements (% w/w of DW), C:N ratio, and N to protein conversion factor in bread waste (BW) samples.

BW sample	BW type	Nutritional values (% w/w of DW)			DW, % w/w 105°C, 3 days	AC % w/w of DW	Elemental analysis (in DW)			N-to-protein conversion factor
		Carbohydrates % w/w	Lipids % w/w	Proteins % w/w			C % w/w	N % w/w	C:N ratio	
BW-1	Whole wheat	36.2	2.8	10.3	58.8 ± 0.6	3.3 ± 0.1	43.1 ± 0.5	3.0 ± 0.1	14.4 ± 0.2	5.8 ± 0.1
BW-2	Sliced wholemeal bread	25.0	3.1	15.1	53.8 ± 0.5	7.1 ± 0.4	43.8 ± 1.1	4.5 ± 0.0	9.7 ± 0.2	6.2 ± 0.0
BW-3	Light bread	25.5	0.9	13.0	62.8 ± 0.6	4.6 ± 0.4	39.9 ± 4.1	3.8 ± 0.3	10.5 ± 0.1	5.5 ± 0.3
BW-4	Sliced black bread	56.5	2.5	10.3	61.3 ± 0.8	2.9 ± 0.3	41.8 ± 0.1	2.1 ± 0.0	19.9 ± 0.1	7.9 ± 0.1
BW-5	Light bite pita	31.5	0.0	10.2	64.5 ± 0.7	4.0 ± 0.0	43.6 ± 0.4	3.2 ± 0.0	13.6 ± 0.1	4.9 ± 0.1
BW-6	Flax bread	4.0	16.4	27.5	64.1 ± 0.4	3.7 ± 0.3	52.0 ± 1.6	7.3 ± 0.2	7.1 ± 0.2	5.9 ± 0.2
BW-7	Sliced uniform white	51.2	1.9	9.5	61.0 ± 0.7	1.6 ± 0.1	42.6 ± 0.9	2.4 ± 0.1	17.8 ± 0.3	6.5 ± 0.1
BW-8	Cereal bread	25.4	25.4	12.2	59.7 ± 0.4	5.7 ± 1.2	42.7 ± 0.9	3.0 ± 0.2	14.2 ± 0.3	6.8 ± 0.2
BW-9	White pita-1	55.7	1.2	9.1	65.6 ± 0.6	3.4 ± 0.6	42.0 ± 1.3	2.1 ± 0.1	20.0 ± 0.6	6.6 ± 0.1
BW-10	Rye flour pita	46.0	2.0	11.2	54.5 ± 0.5	3.2 ± 0.1	42.4 ± 0.2	3.0 ± 0.2	14.1 ± 0.1	6.9 ± 0.2
BW-11	Wholemeal pita	58.0	2.0	10.0	58.1 ± 0.4	3.5 ± 0.2	42.2 ± 0.3	3.0 ± 0.0	14.1 ± 0.1	5.7 ± 0.1
BW-12	Light pita	43.0	0.7	9.7	56.9 ± 0.3	4.4 ± 1.4	42.9 ± 0.8	3.2 ± 0.1	13.4 ± 0.3	5.3 ± 0.1
BW-13	White Pita-2	55.7	1.2	5.8	58.8 ± 0.4	3.3 ± 0.6	39.7 ± 0.2	1.7 ± 0.0	23.4 ± 0.1	5.8 ± 0.1

The BW samples were dried at 105°C for 3 days. Mean ± SD, *n* = 3.

### Optimization of biomass production from BW by *Haloferax mediterranei*

**Figure 1:**
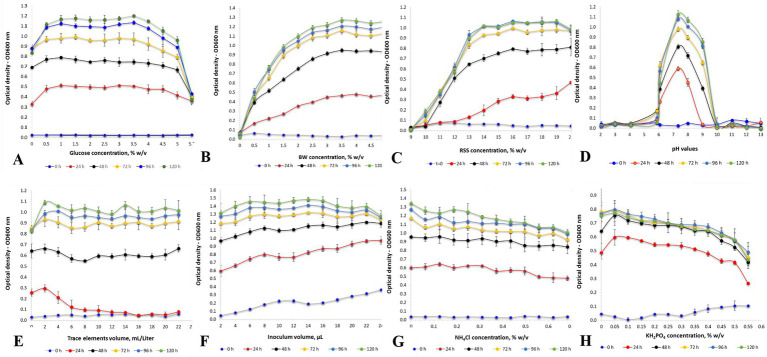
**(A)** Effect of different glucose concentrations on the optical density (OD_600_) of *H. mediterranei* culture. *H. mediterranei* was cultivated using a 96-well plate, 198 μL medium, 2 μL inoculum, 5 replicates for each treatment, 3 triplicates for each blank, pH 7.5, yeast extract 0.5% w/v, red sea salt 17.5% w/v, 150 rpm, at 42°C. **(B)** Effect of different concentrations of bread waste (BW). **(C)** Effect of different concentrations of red sea salt. **(D)** Effect of different pH values. **(E)** Effect of different trace element concentrations. **(F)** Effect of different inoculum volumes. **(G)** Effect of different NH_4_Cl concentrations. **(H)** Effect of different KH_2_PO_4_ concentrations.

The current study extensively investigated the key factors that impact the growth of *H. mediterranei* under various challenging conditions (Figure 1; Table 2). Specifically, the effect of the concentration of red sea salt (9–20.0%w/v), trace elements (0–2.4% w/v), NH_4_Cl (0–0.7% w/v), KH_2_PO_4_ (0–0.66% w/v), glucose (0–5.5% w/v), and BW hydrolysate (DW of 0.5–5.5% w/v), as well as pH variations (2–13) were investigated. The enzymatic hydrolysis of BW samples and their mixtures was carried out with liquefaction by *α*-amylase, saccharification by amyloglucoamylase, and protein hydrolysis by endopeptidase alcalase. *H. mediterranei* cultivation was carried out in a 96-well plate at 42°C and 150 rpm for 120 h.

**Table 2:** Effect of *H. mediterranei* cultivation conditions (glucose concentration, bread waste-BW concentration, red sea salt-RSS concentration, pH, trace elements concentration, and NH_4_Cl concentration) on the specific growth rate (*μ*, h^−1^), doubling time (*d.t.*, h), cell dry biomass (DW, mg mL^−1^), PHBV yield (% w/w), protein yield (% w/w), PHBV content (mg mL^−1^), protein content (mg mL^−1^), and protein/PHBV ratio.

Variable (fermentation conditions)	Value	*μ*, h^−1^	*d.t.*, h	DW, mg mL^−1^	PHBV, % w/w	Protein % w/w	PHBV, mg mL^−1^	Protein mg mL^−1^	Protein/PHBV ratio
**Glucose concentration** (% w/v) (2 μL inoculum, pH = 7.5, red sea salt 17.5% w/v, 150 rpm, 42°C)	0.0	0.108 ± 0.009	6.4 ± 0.5	7.2 ± 0.3	11.9 ± 0.1	30.6 ± 0.5	0.86 ± 0.5	2.2 ± 0.4	2.6 ± 0.1
	0.5	0.122 ± 0.009	5.7 ± 0.4	8.8 ± 0.1	14.5 ± 0.2	22.4 ± 0.6	1.3 ± 0.4	1.9 ± 0.2	1.5 ± 0.2
	1.0	0.125 ± 0.003	5.6 ± 0.1	7.2 ± 0.5	18.1 ± 0.2	22.3 ± 0.7	1.3 ± 0.3	1.6 ± 0.3	1.2 ± 0.1
	1.5	0.122 ± 0.004	5.7 ± 0.2	13.6 ± 0.3	15.9 ± 0.1	22.6 ± 0.8	2.2 ± 0.6	3.0 ± 0.2	1.4 ± 0.1
	2.0	0.133 ± 0.006	5.2 ± 0.2	5.9 ± 0.2	23.0 ± 0.3	24.3 ± 0.2	1.4 ± 0.3	1.4 ± 0.5	1.1 ± 0.1
	2.5	0.129 ± 0.006	5.4 ± 0.3	8.9 ± 0.2	11.3 ± 0.9	18.4 ± 0.4	1.0 ± 0.2	1.6 ± 0.1	1.6 ± 0.2
	3.0	0.132 ± 0.002	5.2 ± 0.1	8.9 ± 0.4	16.5 ± 0.7	26.2 ± 0.1	1.5 ± 0.4	2.3 ± 0.3	1.6 ± 0.1
	3.5	0.124 ± 0.002	5.6 ± 0.1	9.9 ± 0.6	15.4 ± 0.8	19.7 ± 0.2	1.5 ± 0.3	2.0 ± 0.4	1.3 ± 0.0
	4.0	0.125 ± 0.012	5.5 ± 0.5	10.2 ± 0.7	15.8 ± 0.4	18.9 ± 0.1	1.6 ± 0.2	1.9 ± 0.6	1.2 ± 0.2
	4.5	0.130 ± 0.010	5.3 ± 0.4	6.3 ± 0.2	11.2 ± 0.5	14.6 ± 0.3	0.7 ± 0.4	0.9 ± 0.1	1.3 ± 0.1
	5.0	0.116 ± 0.010	6.0 ± 0.5	8.8 ± 0.1	16.4 ± 0.7	19.8 ± 0.4	1.4 ± 0.1	1.7 ± 0.2	1.2 ± 0.1
	5.5	0.108 ± 0.010	6.4 ± 0.6	14.1 ± 0.3	11.9 ± 0.9	30.6 ± 0.6	1.4 ± 0.1	4.3 ± 0.5	2.6 ± 0.1
**BW concentration** (% w/v) (2 μL inoculum, pH = 7.5, red sea salt 17.5% w/v, 150 rpm, 42°C)	0.0	0.019 ± 0.004	35.7 ± 7.5	0.0 ± 0.0	n.d.	n.d.	n.d.	n.d.	n.d.
	0.5	0.040 ± 0.012	17.5 ± 5.4	2.5 ± 0.1	19.9 ± 1.0	21.3 ± 1.1	0.5 ± 0.0	0.5 ± 0.0	1.1 ± 0.1
	1.0	0.060 ± 0.001	11.6 ± 0.2	4.6 ± 0.2	18.2 ± 0.9	19.5 ± 1.0	0.8 ± 0.0	0.9 ± 0.0	1.1 ± 0.1
	1.5	0.074 ± 0.010	9.4 ± 1.2	8.2 ± 0.4	17.5 ± 0.8	20.1 ± 1.0	1.4 ± 0.1	1.6 ± 0.1	1.2 ± 0.1
	2.0	0.087 ± 0.004	8.0 ± 0.4	10.7 ± 0.6	17.8 ± 0.8	20.0 ± 1.0	1.9 ± 0.2	2.1 ± 0.1	1.1 ± 0.1
	2.5	0.099 ± 0.007	7.0 ± 0.5	9.8 ± 0.5	10.6 ± 0.5	14.2 ± 0.7	1.0 ± 0.0	1.4 ± 0.1	1.4 ± 0.2
	3.0	0.110 ± 0.016	6.3 ± 0.9	12.6 ± 0.6	21.4 ± 1.1	24.4 ± 1.2	2.7 ± 0.2	3.1 ± 0.2	1.1 ± 0.1
	3.5	0.117 ± 0.014	5.9 ± 0.7	13.6 ± 0.6	16.3 ± 0.8	19.2 ± 0.9	2.2 ± 0.1	2.6 ± 0.2	1.2 ± 0.1
	4.0	0.103 ± 0.008	6.7 ± 0.5	15.1 ± 0.7	17.3 ± 0.9	22.7 ± 1.2	2.6 ± 0.2	3.4 ± 0.2	1.3 ± 0.2
	4.5	0.105 ± 0.010	6.6 ± 0.6	11.7 ± 0.6	19.9 ± 1.0	28.0 ± 1.4	2.3 ± 0.1	3.3 ± 0.2	1.4 ± 0.2
	5.0	0.103 ± 0.004	6.7 ± 0.2	11.7 ± 0.6	15.8 ± 0.8	16.7 ± 0.8	1.8 ± 0.1	1.9 ± 0.1	1.1 ± 0.1
	5.5	0.105 ± 0.009	6.6 ± 0.6	5.0 ± 0.3	17.1 ± 0.8	14.9 ± 0.7	0.9 ± 0.0	0.7 ± 0.0	0.9 ± 0.1
**RSS concentration** (% w/v) (2 μL inoculum, pH = 7.5, bread waste 2% w/v, 150 rpm, 42°C)	9	0.003 ± 0.001	213.8 ± 39.4	0.0 ± 0.0	n.d.	n.d.	n.d.	n.d.	n.d.
	10	0.004 ± 0.001	193.0 ± 65.4	0.0 ± 0.0	n.d.	n.d.	n.d.	n.d.	n.d.
	11	0.004 ± 0.000	160.9 ± 4.8	0.0 ± 0.0	n.d.	n.d.	n.d.	n.d.	n.d.
	12	0.007 ± 0.000	103.2 ± 1.4	0.8 ± 0.0	22.2 ± 1.1	14.9 ± 0.7	0.2 ± 0.0	0.1 ± 0.0	0.7 ± 0.0
	13	0.030 ± 0.003	22.9 ± 2.3	2.0 ± 0.1	33.7 ± 1.7	26.1 ± 1.3	0.7 ± 0.0	0.5 ± 0.0	0.8 ± 0.1
	14	0.047 ± 0.012	14.8 ± 3.9	3.3 ± 0.2	24.1 ± 1.2	32.7 ± 1.6	0.8 ± 0.1	1.1 ± 0.1	1.4 ± 0.1
	15	0.063 ± 0.006	11.0 ± 1.1	3.4 ± 0.2	31.7 ± 1.6	23.7 ± 1.2	1.1 ± 0.1	0.8 ± 0.1	0.8 ± 0.0
	16	0.079 ± 0.004	8.7 ± 0.5	6.1 ± 0.3	18.7 ± 0.9	22.0 ± 1.1	1.1 ± 0.1	1.3 ± 0.1	1.2 ± 0.1
	17	0.084 ± 0.006	8.3 ± 0.6	6.6 ± 0.3	20.3 ± 1.0	21.5 ± 1.1	1.3 ± 0.1	1.4 ± 0.2	1.1 ± 0.1
	18	0.077 ± 0.008	9.1 ± 1.0	5.6 ± 0.3	21.7 ± 1.1	21.7 ± 1.1	1.2 ± 0.1	1.2 ± 0.1	1.0 ± 0.1
	19	0.084 ± 0.014	8.3 ± 1.4	6.9 ± 0.4	20.8 ± 1.0	23.4 ± 1.2	1.4 ± 0.2	1.6 ± 0.2	1.1 ± 0.1
	20	0.097 ± 0.002	7.2 ± 0.2	12.3 ± 0.6	20.0 ± 1.0	20.5 ± 1.0	2.5 ± 0.3	2.5 ± 0.3	1.0 ± 0.1
**pH** (2 μL inoculum, red sea salt 17.5% w/v, bread waste 2% w/v, 150 rpm, 42°C)	2.1	n.d.	n.d.	n.d.	n.d.	n.d.	n.d.	n.d.	n.d.
	3.0	n.d.	n.d.	n.d.	n.d.	n.d.	n.d.	n.d.	n.d.
	4.1	n.d.	n.d.	n.d.	n.d.	n.d.	n.d.	n.d.	n.d.
	5.9	0.022 ± 0.004	32.2 ± 5.8	1.7 ± 0.1	17.0 ± 0.8	18.5 ± 0.9	0.3 ± 0.0	0.3 ± 0.0	1.1 ± 0.1
	6.1	0.086 ± 0.002	8.1 ± 0.2	7.2 ± 0.4	22.5 ± 1.1	22.4 ± 1.1	1.6 ± 0.2	1.6 ± 0.2	1.0 ± 0.1
	7.3	0.133 ± 0.005	5.2 ± 0.2	8.3 ± 0.4	19.4 ± 1.0	21.7 ± 1.1	1.6 ± 0.2	1.8 ± 0.2	1.1 ± 0.1
	8.0	0.094 ± 0.008	7.4 ± 0.6	3.0 ± 0.2	29.3 ± 1.5	19.7 ± 1.0	0.9 ± 0.1	0.6 ± 0.0	0.7 ± 0.0
	9.0	0.009 ± 0.001	78.5 ± 5.4	1.6 ± 0.1	5.6 ± 0.3	9.4 ± 0.4	0.1 ± 0.0	0.1 ± 0.0	1.7 ± 0.2
	10.0	n.d.	n.d.	n.d.	n.d.	n.d.	n.d.	n.d.	n.d.
	11.0	n.d.	n.d.	n.d.	n.d.	n.d.	n.d.	n.d.	n.d.
	12.0	n.d.	n.d.	n.d.	n.d.	n.d.	n.d.	n.d.	n.d.
	13.0	n.d.	n.d.	n.d.	n.d.	n.d.	n.d.	n.d.	n.d.
**Trace elements concentration** (% v/v) (2 μL inoculum, pH = 7.5, red sea salt 17.5% w/v, bread waste 2% w/v, 150 rpm, 42°C)	0	0.089 ± 0.012	7.8 ± 1.0	4.6 ± 0.3	32.8 ± 1.6	32.6 ± 1.6	1.5 ± 0.2	1.5 ± 0.2	1.0 ± 0.1
	2	0.084 ± 0.009	8.2 ± 0.9	2.4 ± 0.1	28.2 ± 1.4	29.0 ± 1.5	0.7 ± 0.1	0.7 ± 0.1	1.0 ± 0.1
	8	0.038 ± 0.007	18.2 ± 3.4	2.8 ± 0.2	13.5 ± 0.7	11.0 ± 0.5	0.4 ± 0.0	0.3 ± 0.0	0.8 ± 0.1
	10	0.024 ± 0.010	29.4 ± 13.1	8.4 ± 0.4	30.5 ± 1.5	40.4 ± 1.9	2.6 ± 0.3	3.4 ± 0.3	1.3 ± 0.2
	12	0.016 ± 0.004	42.5 ± 10.7	9.3 ± 0.5	28.3 ± 1.4	30.8 ± 1.6	2.6 ± 0.3	2.9 ± 0.3	1.1 ± 0.1
	14	0.011 ± 0.000	60.3 ± 1.9	7.6 ± 0.4	27.3 ± 1.4	28.0 ± 1.4	2.1 ± 0.2	2.1 ± 0.2	1.0 ± 0.1
	16	0.008 ± 0.002	86.1 ± 25.6	4.4 ± 0.2	29.3 ± 1.5	30.0 ± 1.5	1.3 ± 0.2	1.3 ± 0.1	1.0 ± 0.1
	18	0.006 ± 0.001	116.1 ± 18.6	8.0 ± 0.4	29.3 ± 1.5	30.3 ± 1.5	2.3 ± 0.3	2.4 ± 0.2	1.0 ± 0.1
	20	0.015 ± 0.006	45.2 ± 19.1	5.7 ± 0.3	34.9 ± 1.7	33.4 ± 1.2	2.0 ± 0.2	1.9 ± 0.2	1.0 ± 0.1
	22	0.012 ± 0.002	58.8 ± 10.4	8.3 ± 0.4	25.6 ± 1.3	33.0 ± 1.2	2.1 ± 0.2	2.7 ± 0.3	1.3 ± 0.2
**NH**_**4**_**Cl concentration** (% w/v) (2 μL inoculum, pH = 7.5, red sea salt 17.5% w/v, bread waste 2% w/v, 150 rpm, 42°C)	0	0.070 ± 0.002	9.9 ± 0.3	11.6 ± 0.6	22.0 ± 1.1	22.0 ± 1.1	2.5 ± 0.2	2.6 ± 0.3	1.0 ± 0.1
	0.062	0.062 ± 0.004	11.2 ± 0.7	7.4 ± 0.3	22.2 ± 1.1	19.8 ± 1.0	1.6 ± 0.1	1.5 ± 0.1	0.9 ± 0.1
	0.124	0.059 ± 0.002	11.7 ± 0.4	12.9 ± 0.6	20.2 ± 1.0	19.5 ± 1.0	2.6 ± 0.2	2.5 ± 0.2	1.0 ± 0.1
	0.186	0.073 ± 0.004	9.5 ± 0.5	14.4 ± 0.7	22.2 ± 1.1	22.9 ± 1.1	3.2 ± 0.3	3.3 ± 0.3	1.0 ± 0.1
	0.248	0.069 ± 0.001	10.0 ± 0.0	15.3 ± 0.6	20.4 ± 1.0	21.6 ± 1.1	3.1 ± 0.3	3.3 ± 0.3	1.1 ± 0.1
	0.310	0.075 ± 0.001	9.3 ± 0.5	19.9 ± 1.0	18.8 ± 0.9	20.3 ± 1.0	3.7 ± 0.4	4.0 ± 0.4	1.1 ± 0.1
	0.372	0.073 ± 0.004	9.5 ± 0.5	12.5 ± 0.6	22.9 ± 1.2	22.1 ± 1.1	2.9 ± 0.3	2.8 ± 0.3	1.0 ± 0.1
	0.434	0.075 ± 0.004	9.3 ± 0.5	5.6 ± 0.3	19.2 ± 0.9	22.2 ± 1.1	1.1 ± 0.1	1.2 ± 0.1	1.2 ± 0.1
	0.496	0.057 ± 0.004	12.1 ± 1.0	8.1 ± 0.4	23.8 ± 1.2	21.6 ± 1.1	1.9 ± 0.2	1.7 ± 0.2	0.9 ± 0.1
	0.558	0.062 ± 0.001	11.4 ± 0.4	7.0 ± 0.3	23.7 ± 1.2	24.3 ± 1.2	1.7 ± 0.2	1.7 ± 0.2	1.0 ± 0.1
	0.620	0.074 ± 0.008	9.4 ± 1.1	3.2 ± 0.2	24.6 ± 1.3	22.6 ± 1.2	0.8 ± 0.1	0.7 ± 0.1	0.9 ± 0.1
	0.682	0.062 ± 0.003	11.3 ± 0.6	18.9 ± 0.9	19.7 ± 1.0	22.2 ± 1.1	3.7 ± 0.3	4.2 ± 0.4	1.1 ± 0.1

Mean ± SD, *n* = 5.

n.d., not detected.

#### Effect of BW concentration on cell biomass production

Optimized archaea growth was observed using a BW concentration range of 2.5 to 5.5% w/v (Figure 1B). The optimized specific growth rate (*μ*) and doubling time (d.t.) (Rubin et al., 2023) obtained with 3.0% w/v BW were 0.11 ± 0.02 h^−1^ and 6.3 ± 0.9 h, respectively. The composition of the resulting cell biomass was determined by FTIR analysis, which revealed a PHBV content of 21.4 ± 1.1% (w/w) and a protein content of 24.4 ± 1.2% (w/w). Montemurro et al. previously demonstrated the suitability of enzymatically hydrolyzed BW, supplemented with seawater, as a substrate for bioplastic production by fermenting *H. mediterranei* (Montemurro et al., 2022).

#### Effect of red sea salt concentration on *Haloferax mediterranei* biomass production

Different concentrations of red sea salt (Figure 1C) were examined in the cultivation of *H. mediterranei*. No statistically significant differences in the OD_600_ were observed for red sea salt concentrations in the range of 15.0 to 20.0% w/v. On the other hand, at 10.0% w/v, there was no observed growth, and a delayed growth response was detected at lower concentrations of red sea salt. The optimized values of 0.097 ± 0.002 h^−1^ for μ and 7.2 ± 0.2 h for d.t. were achieved using 20.0% w/v red sea salt. The cell biomass produced under these conditions contained 20.0 ± 1.0% (w/w) of PHBV and 20.5 ± 1.0% (w/w) of protein. These findings align with the Matarredona et al. study, which reported an optimum growth rate at sea salt concentrations between 10.0 and 32.5% w/v (Matarredona et al., 2021). *H. mediterranei* requires a minimum of 10.0% w/v salt for growth and can thrive in its natural environment with salt concentrations above 20.0% w/v. This indicates the remarkable ability of *H. mediterranei* to withstand extreme salinity levels, and it has efficient osmoregulatory mechanisms that most likely allow it to maintain an adequate cellular water balance and grow effectively under varying salinity conditions.

#### Effect of pH on biomass production

A wide range of medium pH values, from 2 to 13, was applied in cultivating *H. mediterranei*. It was observed that *H. mediterranei* could not survive when the pH of the cultivation medium was lower than 5.9 or higher than 9. However, it exhibited its most significant growth at a pH of 7.3 with a μ value of 0.133 ± 0.005 h^−1^, a d.t. value of 5.2 ± 0.2 h, a PHBV value of 19.4 ± 1.0% w/w, and a protein concentration of 21.7 ± 1.1% w/w (Figure 1D). The results were similar to those of Matarredona et al., where the optimum growth of *H. mediterranei* was achieved at pH 7.25 (Matarredona et al., 2021).

#### Effect of the BW type on cell biomass production

**Figure 2:**
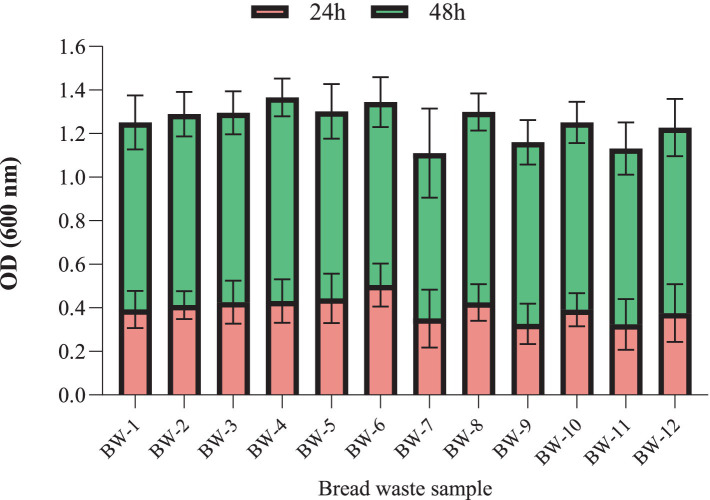
Effect of different bread waste samples (BW-1 to BW-12) (2.0% w/v) on the optical density (OD_600_) of *H. mediterranei* culture. *H. mediterranei* was cultivated using a 96-well plate, 198 μL medium, 2 μL inoculum, 5 replicates for each treatment, triplicate for each blank, pH 7.5, red sea salt 17.5% w/v, 150 rpm, at 42°C, for 24 h (red) and 48 h (green).

A two-way ANOVA study with repeated measures was performed to examine the effect of BW type on *H. mediterranei* growth (Figure 2). Following this analysis, a Tukey multiple comparison test was conducted to pinpoint significant differences between the group means; it revealed a significant effect of time, e.g., 24 h compared to 48 h, *F* (1, 84) = 6,819, *p* < 0.0001. Moreover, it was observed that there was no significant effect of the various bread samples on the growth of *H. mediterranei*, as indicated by the non-significant results of the statistical analysis (*F* (11, 84) = 1.147, *p* = 0.3362). Subsequently, a Tukey multiple comparisons test was conducted to investigate pairwise differences between the BW sample levels. The results revealed no significance (*p* > 0.05) between all BW samples.

In summary, the study highlighted the significance of substrate concentration, specifically the enzymatic hydrolysate of BW, with an optimum performance observed at 3.0% w/v of BW. Salinity, represented by red sea salt concentrations of 15.0 to 20.0% w/v, substantially influenced the growth and provided the most favorable conditions. Moreover, pH emerged as a vital factor, and neutral values at around 7.3 resulted in the highest growth rates. Furthermore, the study’s findings enable the use of different BW mixtures, effectively minimizing the impact of BW composition fluctuations on the quality of the resulting archaea cell biomass.

### Batch cultivation of *Haloferax mediterranei* for biomass production

In the batch cultivation of *H. mediterranei* under optimized conditions, i.e., pH 7.3, at 42°C, and 150 rpm for 3 days, a mixture of 3.0% w/v of BW hydrolysate and 20.0% w/v of red sea salt was found to produce a maximum cell biomass concentration of 8.0 ± 0.1 g L^−1^ with a productivity of 11.1 mg L^−1^ h^−1^. The biomass contained 36.0 ± 6.3% (w/w) of PHBV. The stoichiometry for the fermentation of *H. mediterranei* on BW was obtained by a CHNS elemental mass balance, revealing a chemical formula of [C_355_H_660_N_23_S_2.6_O_297_] for BW and [C_241_H_408_N_27_S_3_O_390_] for the cell biomass, as presented in Equation 7:


(7) $$1.17\;\left[BW\right]+286\;O_{2}\to 1\;\left[\mathrm{Cell biomass}\right]+174\;{CO}_{2}+182\;H_{2}O$$

During fermentation, *H. mediterranei* utilizes BW as a carbon and nitrogen source, along with oxygen (O_2_), to grow and produce biomass. This metabolic process generates carbon dioxide (CO_2_) and water (H_2_O) as byproducts. The reaction’s stoichiometry ensures a balanced distribution of all elements (C, H, N, S, and O). CO_2_ was determined by comparing the C content in the BW hydrolysate to that in the produced biomass. The difference in C atoms for the CO_2_ generated, and when scaled to 1 mole of cell biomass, 174 moles of CO_2_ are produced, ensuring a complete C balance. More in detail, the BW hydrolysate has a chemical composition represented by [C_355_H_660_N_23_S_2.6_O_297_], meaning that each unit of BW contains 355 moles of C atoms, 660 moles of H atoms, 23 moles of N atoms, 2.6 moles of S atoms, and 297 moles of O atoms. Similarly, the cell biomass produced from fermentation has the formula [C_241_H_408_N_27_S_3_O_390_]. The total amount of C atoms in the BW hydrolysate is either incorporated into the cell biomass or released as CO_2_. Since each mole of BW contains 355 moles of C atoms, and the resulting biomass contains 241 moles, the remaining 114 moles of C are released as CO₂. Therefore, for every 1 mole of BW hydrolysate consumed, 114 moles of CO_2_ are produced. The final coefficient of CO_2_ (174 moles) is determined in the equation by scaling the balance to 1 mole of cell biomass. The O_2_ provided during the fermentation also contributes to the complete oxidation of C into CO_2_. O_2_ and H_2_O are also accounted for in order to balance the equation completely based on H and O conservation.

Examples of cell biomass and PHA production by *H. mediterranei* utilizing various industrial and agricultural wastes compared to this study are presented in Table 3. These results show that PHA contents range from 6 to 57% (w/w) compared to 36.0% in the current study.

**Table 3:** Literature examples of industrial and agricultural waste bioconversion to cell biomass and PHA by *H. mediterranei.*

Waste	Cell biomass g L^−1^	PHA (% w/w)	References
Bread waste	8	36	This study
Bread waste	3–6	24	Montemurro et al. (2022)
Rice bran	63–140	27–56	Huang et al. (2006)
Cheese whey	5.7	29–65	Pais et al. (2016)
Olive mill wastewater	0.46	43	Alsafadi and Al-Mashaqbeh (2017)
Macroalgal biomass	3.8	57	Ghosh et al. (2019)
Ricotta cheese exhausted whey	10–18	6–10	Raho et al. (2020)
Date palm fruit waste	12.8	24	Alsafadi et al. (2020)

### Composition of the archaea cell biomass

The ash content in the *H. mediterranei* biomass was 20.2% w/w, which surpasses that of several protein sources, including yeast (5–9.5% w/w), bacteria (3–7% w/w), plants (0.5–1.6% w/w), milk (5.8% w/w), beef (4% w/w), and eggs (3.9% w/w) (Nalage et al., 2016), but it was lower than that of seaweed (30–40% w/w) (Sonchaeng et al., 2023) (Table 4). This notable mineral content of *H. mediterranei* is due to its high salinity habitat.

**Table 4:** Examples of ash content (%) obtained from various sources.

Source	Ash content %
Archaea (*H. mediterranei*)	20.2
Yeast	5–9.5
Plants	0.5–1.6
Milk	5.8
Bacteria	3–7
Beef	4
Eggs	3.9
Seaweed	30–40

The CHNS elemental analysis of the cell biomass revealed 28.9 ± 4.9% carbon, 3.8 ± 0.6% nitrogen, and 0.9 ± 0.2% sulfur. The C:N ratio was 7.6 ± 1.3, and the N content determined by the Kjeldahl method was 4.0 ± 0.3%, implying a protein-rich composition.

The total macroelements in cell biomass were 10.91 ± 0.13 g kg^−1^ and mainly consisted of Na 7.05 ± 0.36 g kg^−1^, K 1.04 ± 0.18 g kg^−1^, P 0.91 ± 0.04 g kg^−1^, Mg 0.85 ± 0.05 g kg^−1^, S 0.73 ± 0.10 g kg^−1^, and Ca 0.34 ± 0.02 g kg^−1^. The total trace elements in cell biomass were 91.5 ± 0.9 mg kg^−1^, mainly comprising Fe, Si, Al, Li, B, and Cu.

Archaea cell biomass had a low lipid content of 0.93 ± 0.02 g kg^−1^; it mainly consisted of palmitic acid, stearic acid, 1-hexadecanol, and 1-tetracosanol, as well as a low level of carbohydrate content, 3.0 ± 0.2 g kg^−1^; it consisted of arabinose, galactose, and glucose.

The amino acid profile of archaea cell biomass (Table 5) consists of 17 amino acids. Tryptophan remained undetectable under the analysis conditions, whereas both asparagine and glutamine were incorporated within the measured values of aspartic acid and glutamic acid, respectively. The archaea cell biomass comprised 358.0 ± 3.9 g kg^−1^ of amino acids, 146.7 ± 4.8 g kg^−1^ of essential amino acids (Hou and Wu, 2018), 211.3 ± 1.0 g kg^−1^ of non-essential amino acids, and 71.2 ± 0.7 g kg^−1^ of branch chain amino acids. According to the Food and Agriculture Organization of the United Nations (FAO, 2010), the total amino acids in rolled oats 101 g kg^−1^, lentils 16.9 g kg^−1^, wheat 86 g kg^−1^, peas 170 g kg^−1^, and kidney beans 165 g kg^−1^, are lower than those of archaea cell biomass 358 g kg^−1^, whereas casein 775 g kg^−1^, has a higher amount of total amino acids. In addition, the essential amino acids in rolled oats 37 g kg^−1^, lentils 67 g kg^−1^, wheat 28 g kg^−1^, peas 68 g kg^−1^, and kidney beans 70 g kg^−1^, are lower than those in archaea cell biomass 146.7 g kg^−1^, whereas casein 347 g kg^−1^, has a higher amount of essential amino acids.

**Table 5:** Amino acid profile.

No.	Amino acid	Amount (g kg^−1^)
1	Alanine	23.3 ± 0.2
2	Arginine	24.9 ± 0.3
3	Aspartic acid	45.5 ± 0.2
4	Cysteine	2.9 ± 0.1
5	Glutamic acid	55.7 ± 0.4
6	Glycine	18.1 ± 0.3
7	Proline	13.2 ± 1.2
8	Serine	13.9 ± 0.8
9	Tyrosine	13.7 ± 0.3
10	Histidine	9.2 ± 0.1
11	Isoleucine	16.8 ± 0.2
12	Leucine	26.9 ± 0.1
13	Lysine	15.9 ± 0.7
14	Methionine	7.6 ± 0.2
15	Phenylalanine	19.0 ± 2.2
16	Threonine	23.8 ± 1.3
17	Tryptophan	n.d.
18	Valine	27.5 ± 0.4
	Total non-essential amino acids	211.3 ± 1.0
	Total essential amino acids	146.7 ± 4.8
	Total amino acids	358.0 ± 3.9
	*In vitro* digestibility	0.91 ± 0.02
	Limiting amino acid	L-Lysine
	Limiting amino acid score	0.86 ± 0.01
	Protein digestibility-corrected amino acid score (PDCAAS)	0.78 ± 0.02
	Branch chain amino acids	71.2 ± 0.7

The amount of amino acid (g kg^−1^, mean ± SD, *n* = 3) in *H. mediterranei* cell dry samples. The results were expressed as the means of triplicate ± standard deviation. Tryptophan was not detected.

n.d., not detected.

**Figure 3:**
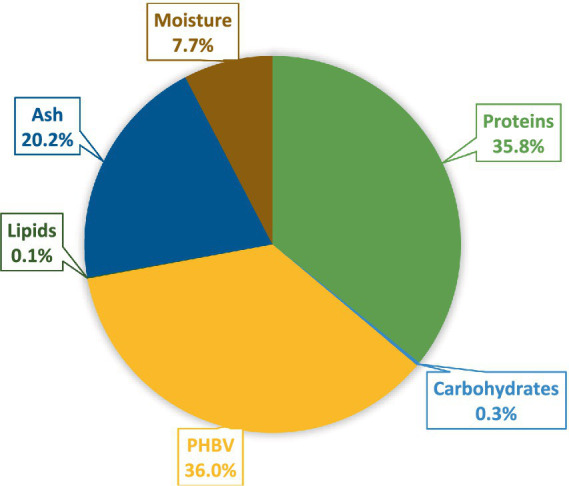
The nutritional profile of cell biomass from *H. mediterranei*: Proteins, PHBV polymer, lipids, carbohydrates, ash, and moisture.

In summary, the archaea cell biomass exhibits substantial protein content and quality. It consists of 358 g kg^−1^ of proteins and 360 g kg^−1^ of PHBV as the major constituents, whereas ash 201.5 g kg^−1^, carbohydrates 3.0 g kg^−1^, lipids 0.93 g kg^−1^, and moisture 76.7 g kg^−1^, contributed to cell biomass’s diverse composition (Figure 3).

### Structural analysis and properties of the archaea cell biomass

**Figure 4:**
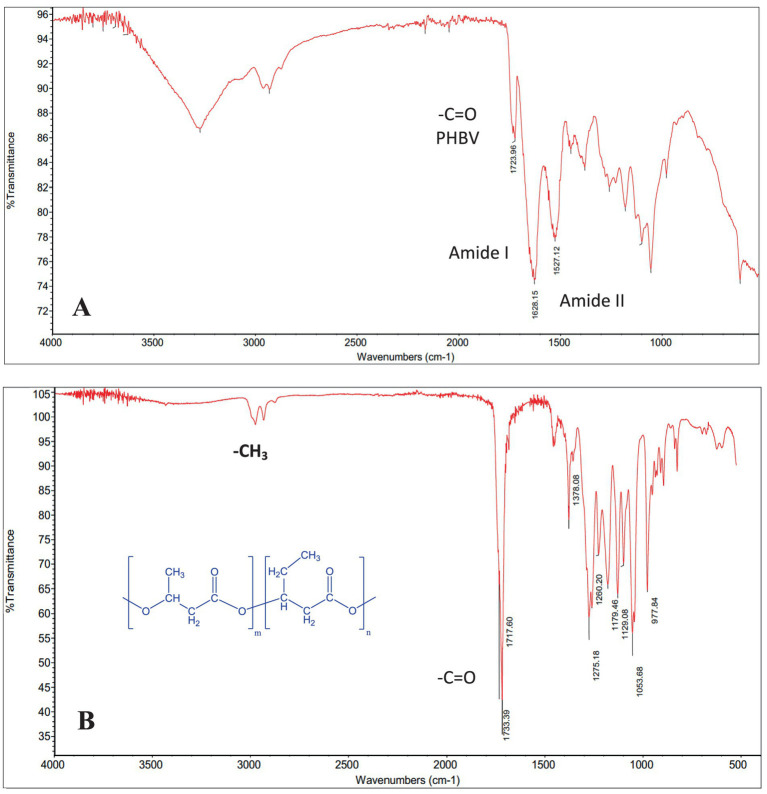
Fourier-Transform Infrared Spectroscopy (FT-IR) spectrum of **(A)** *H. mediterranei* cell biomass cultivated on BW and **(B)** pure PHBV extracted with chloroform.

FTIR analysis of the cell biomass revealed the characteristic vibration band of the ester carbonyl bond (C=O) at 1,724–1,734 cm^−1^ and the stretching band of the C-H bond (CH_3_) at 2,930 cm^−1^, suggesting the presence of PHBV polymer in the cell biomass. The FTIR spectra corresponded to the typical profile of a copolymer PHBV, previously reported in *H. mediterranei* by Simó Cabrera et al. (2024). The 1,628 cm^−1^ and 1,527 cm^−1^ bands correspond to the protein amide I and II vibrations (Figure 4). Other peak frequency assignments, such as 1,179 cm^−1^ and 978 cm^−1^ for C-O-C vibration, 1,054 cm^−1^ for C-O-C and C-C stretching as well as C-O-H bending, were also observed and match the values in the literature (Alsafadi and Al-Mashaqbeh, 2017).

**Figure 5:**
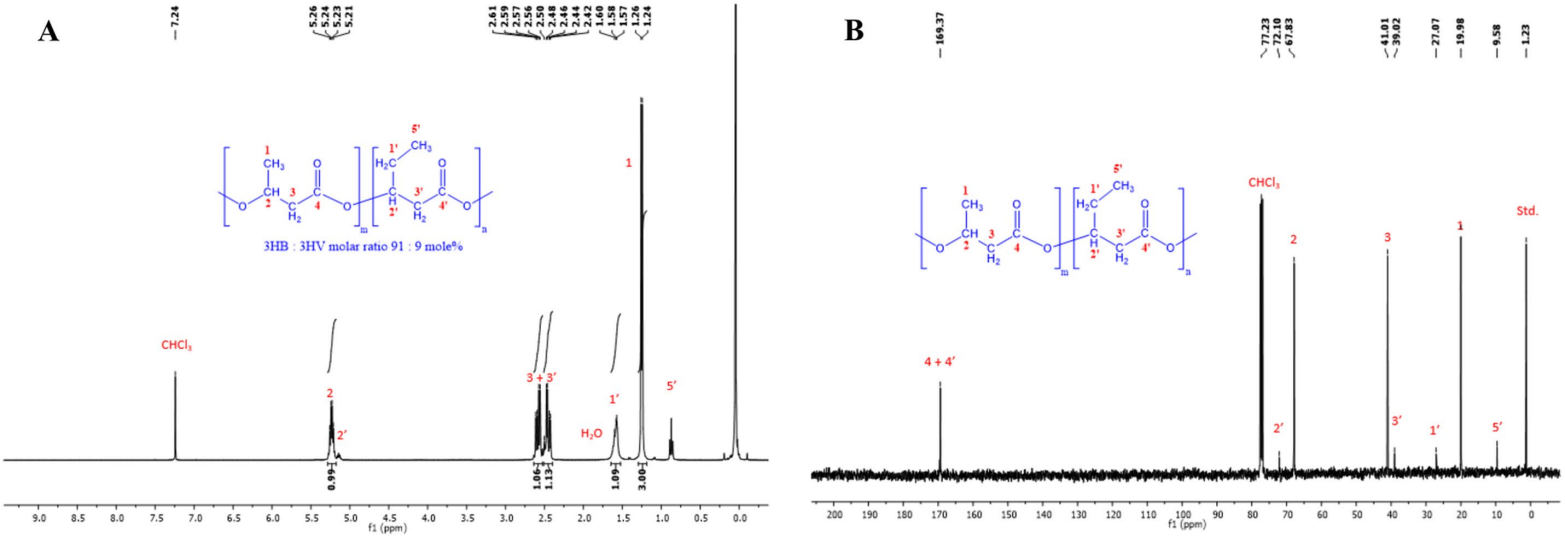
**(A)** Proton nuclear magnetic resonance spectroscopy (^1^H NMR) (400 MHz, CDCl_3_) spectra of PHBV extracted by chloroform from the cell dry mass of *H. mediterranei* cultivated in bread waste (BW). **(B)** Carbon nuclear magnetic resonance spectroscopy (^13^C NMR) (400 MHz, CDCl_3_) spectra of PHBV extracted by chloroform from the cell dry mass of *H. mediterranei* cultivated in bread waste (BW).

The ^13^C NMR spectra of the pure PHBV polymer (Figure 5) displayed 9 singlet signals as follows: at 169.4 ppm for the two **C**=O carbons, 72.1 ppm for the -**C**H-CH_2_CH_3_ carbon, 67.8 ppm for the -**C**H-CH_3_ carbon, 41.0 ppm and 39.0 ppm for the two -**C**H_2_-C=O carbons, 27.1 ppm for the -**C**H_2_-CH_3_ carbon, 20.0 ppm for the -CH-**C**H_3_ carbon, and 9.6 ppm for the -CH_2_-**C**H_3_ carbon. The ^1^H NMR spectra of the PHBV polymer exhibited seven signals as follows: a sextet and quintet at 5.21–5.26 ppm for the -C**H**- hydrogens, two doublets of doublet at 2.42–2.61 ppm for the -C**H**_**2**_-CO- hydrogens, a quintet at 1.58 ppm for the -C**H**_**2**_-CH_3_ hydrogens, a doublet at 1.24–1.26 ppm for the -CH-C**H**_3_, and a triplet at 0.83 ppm for the -CH_2_-C**H**_3_ hydrogens (Ghosh et al., 2019). The ^1^H- and ^13^C-NMR spectra of the extracted PHBV polymer (Figure 5) confirmed the structure of a copolymer of 3-hydroxybutyrate and 3-hydroxyvalerate with a composition of 91:9 mol%, as determined by GC–MS.

**Figure 6:**
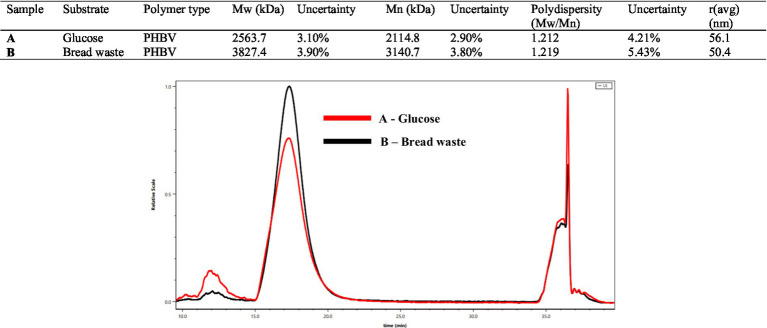
**(A)** Gel permeation chromatography (GPC) of PHBV extracted by chloroform from the cell dry mass of *H. mediterranei* cultivated glucose (red). **(B)** Gel permeation chromatography (GPC) of PHBV extracted by chloroform from the cell dry mass of *H. mediterranei* cultivated in bread waste (BW) (black).

The GPC analysis conducted on the extracted PHBV polymer from *H. mediterranei* cell biomass (Figure 6) revealed that the *M_w_* = 3,827 kDa and *M_n_* = 3,140 kDa values of PHBV produced from BW were notably higher than those of PHBV produced from glucose (*M_w_* = 2,564 kDa, *M_n_* = 2,115 kDa) but had a similar polydispersity index (*PDI = M_w_/M_n_*) of 1.212 and 1.219, respectively, which suggests the production of homogeneous PHBV polymers in both cases. The high *M_w_* value of PHBV reflects its improved mechanical properties, including increased tensile strength and durability. Sato et al. reported that the *M_w_*, *M_n_*, and *PDI* values of PHBV produced by *H. mediterranei* in different media are 1,722–5,280 kDa, 800–3,500 kDa, and 1.6–2.2, respectively (Sato et al., 2021). This study demonstrated that under low concentrations of amino acid sources, 0.1–1 g L^−1^, the *M_w_* = 5,500 kDA and *M_n_* = 3,500 kDa values of the produced PHBV were relatively high, possibly due to the amount of PHBV synthase produced under such conditions.

**Figure 7:**
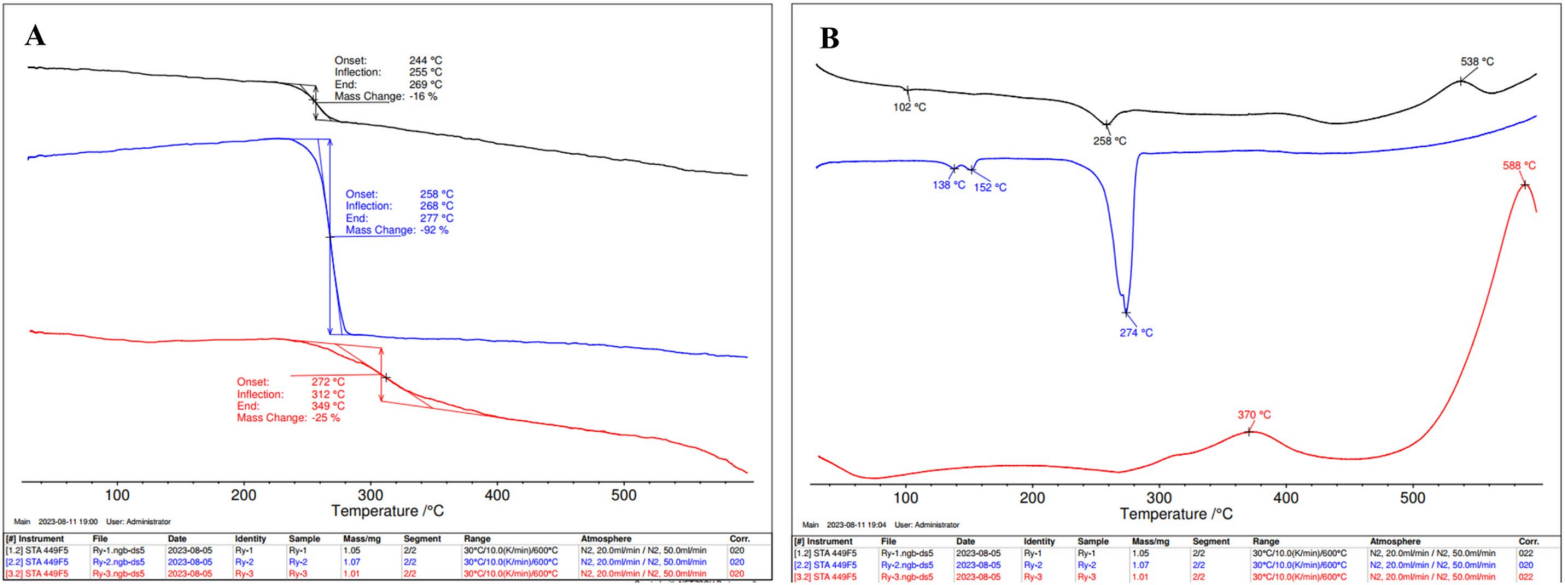
**(A)** Thermo gravimetric analysis (TGA) and **(B)** Differential scanning calorimetry (DSC) of *H. mediterranei* SCP/PHBV (black), PHBV (blue), and protein (red) extracted from *H. mediterranei* cultivated on bread waste (BW).

The thermal stabilities of cell biomass, isolated PHBV, and isolated protein were investigated by TGA and DSC (Figure 7). PHBV exhibited thermal stability with an initial weight decrease at 258°C, *T_d_* at 277°C and *T_m_* at 274°C. The protein underwent thermal degradation starting at 272°C, with a significant structural change at 312°C, *T_d_* at 349°C, and a cross-linking transition at 370°C. Cell biomass experienced thermal degradation at 244°C, featuring a notable structural alteration at 255°C, and it reached a substantial decomposition at 269°C, along with a *T_m_* value of 258°C and a cross-linking point at 538°C.

Archaea protein quality was assessed by evaluating its composition and *in-vitro* protein digestibility-corrected amino acid score (PDCAAS). The calculated parameters for *H. mediterranei* biomass were as follows: the *in-vitro* digestibility was 0.91, the first limiting amino acid (L-lysine) score was 0.86, the essential amino acid ratios ranged from 0.86 to 2.45, the PDCAAS was 0.78, and the crude protein percentage values were 35.8% (Table 5). According to the FAO recommendation (FAO, 2010), the PDCAAS values of high- and excellent-quality protein should be higher than 0.75 and 1.00, respectively (Zeng et al., 2022). Animal protein sources such as casein have PDCAAS values equal to 1 (Qin et al., 2022). Plant protein has lower PDCAAS values that range from 0.39 to 1.00, for instance, and the soy protein PDCAAS value is 0.92 (Rutherfurd et al., 2015), whereas the almond PDCAAS value is 0.39 (Weindl et al., 2020). Edible mycoproteins have a PDCAAS value of 0.35–0.70 (Ahmad et al., 2022), and yeast protein concentrate ranges from 0.82 to 0.90 (Ariëns et al., 2021). Regarding algae, the PDCAAS values range from 0.08 to 0.69 (Zeng et al., 2022). Therefore, archaea biomass derived from *H. mediterranei* has a relatively high protein quality, as indicated by its PDCAAS value (0.78).

### Valorization of bread waste

The carbon footprint of 1,000 g of BW was estimated to be 1,555 g of CO_2_ and 350 mL of CH_4_ emissions (Carlsson and Uldal, 2009). Using BW (10 million tonnes worldwide) in microbial fermentation by *H. mediterranei* to produce cell biomass, rather than allowing it to decompose in landfills, could potentially reduce CH_4_ emissions by 3.5 million tonnes/year while generating 6.5 million tonnes/year of CO_2_. Furthermore, besides the financial and bioresource losses, BW, like any other food waste, causes significant damage to the broader environment by contributing to global warming, acidification, and eutrophication (Ben Rejeb et al., 2022). Therefore, reducing, recycling, or valorizing food waste can potentially save billions of dollars in global economic value, safeguard invaluable bioresources, and prevent the release of millions of tons of greenhouse gasses into the atmosphere.

## Conclusion

This study demonstrated that, despite the variability among BW samples, they were all rich in nitrogen, making them suitable for the production of microbial proteins and biopolymers. Moreover, this study identified critical factors affecting *H. mediterranei* growth, such as pH, red sea salt concentration, and BW concentration. Under optimized conditions, a maximum cell biomass concentration of 8.0 ± 0.1 g L^−1^ was produced. The cell biomass exhibited unique properties, including high protein content and remarkable mineral content due to its high salinity origin. The cell biomass displayed good protein quality, as indicated by its amino acid composition and PDCAAS, making it a promising protein source for various applications.

## Data Availability

The original contributions presented in the study are included in the article/Supplementary material, further inquiries can be directed to the corresponding authors.
