# Supplementary material for: Bioconversion of bread waste into high-quality proteins and biopolymers by fermentation of archaea Haloferax mediterranei
Source: Front Microbiol. 2024 Dec 24;15:1491333. doi: 10.3389/fmicb.2024.1491333 (PMC11703665; doi:10.3389/fmicb.2024.1491333)
Supplement: Supplementary file 1 [file Data_Sheet_1.docx]

**Supplementary Materials**

**Bioconversion of bread waste into high-quality proteins and biopolymers by fermentation of archaea *Haloferax mediterranei***

Razan Unis^1,2⁎^, Rima Gnaim^1,2^ , Mrinal Kashyap^1^, Olga Shamis^3^, Nabeel Gnayem^1,2^, Michael Gozin^3,4,5^, Alexander Liberzon^6^, Jallal Gnaim^2^ and Alexander Golberg^1⁎^

**^1^** Department of Environmental Studies, Porter School of Environment and Earth Sciences, Tel Aviv University, Israel. ^2^ The Triangle Regional R&D Center (TRDC), Kfar Qari 30075, Israel. ^3^ School of Chemistry, Faculty of Exact Sciences, Tel Aviv University, Israel. ^4^ Center for Advanced Combustion Science, Tel Aviv University, Israel. ^5^ Center for Nanoscience and Nanotechnology, Tel Aviv University, Israel. ^6^ School of Mechanical Engineering, Tel Aviv University, Tel Aviv, Israel.

**Correspondence**

Razan Unis, razan.unis@gmail.com; Alexander Golberg, agolberg@ tauex.tau.ac.il

| 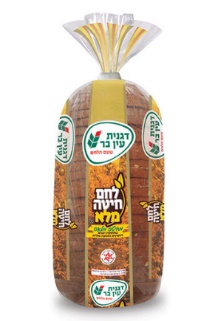  BW-1  **A** | 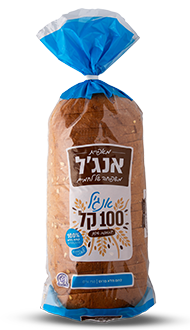  BW-2 | 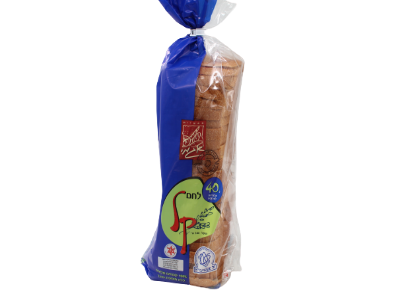  BW-3 | 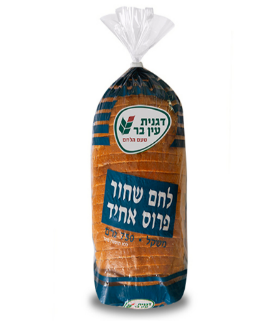  BW-4 | 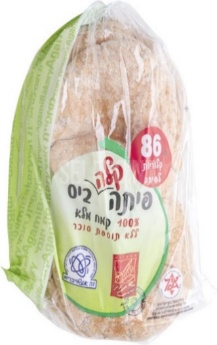  BW-5 | 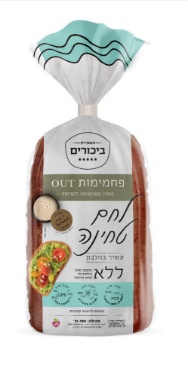  BW-6 | 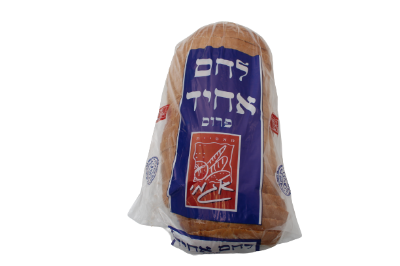  BW-7 |
| --- | --- | --- | --- | --- | --- | --- |
| 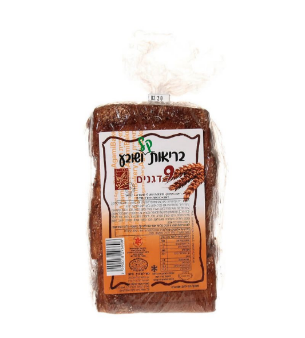  BW-8 | 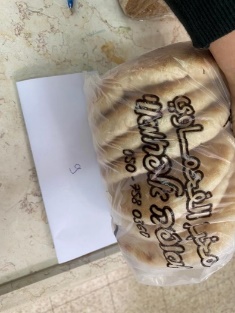  BW-9 | 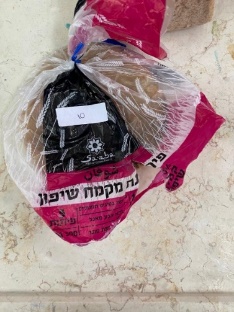  BW-10 | 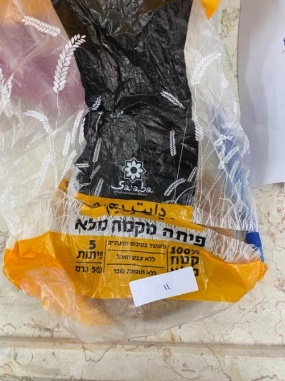  BW-11 | 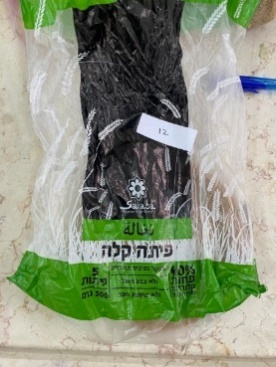  BW-12 | 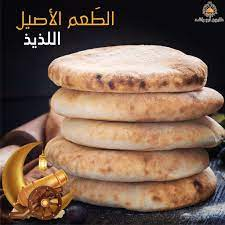  BW-13 |  |

FIGURE S1. Bread samples: BW-1 Whole wheat bread, Deganit Ein Bar, Israel; BW-2 Sliced whole meal bread, Angel - Israel; BW-3 Light bread, Agami, Israel; BW-4 Sliced black bread, Deganit Ein Bar, Israel; BW-5 Light bite pita, Agami, Israel; BW-6 Flax bread - first fruits, Israel; BW-7 Sliced uniform white bread, Israel; BW-8 Cereal bread, Agami, Israel; BW-9 White pita, Alfachamaoui bakery, Israel; BW-10 Rye flour pita, Saaba, Israel; BW-11 Wholemeal pita bread, Saaba, Israel; BW-12 Light pita, Saaba, Israel; BW-13 Light pita, Saaba, Israel.
